# Transcranial magnetic stimulation for individual identification of the best electrode position for a motor imagery-based brain-computer interface

**DOI:** 10.1186/s12984-015-0063-z

**Published:** 2015-08-25

**Authors:** Siegfried Hänselmann, Matthias Schneiders, Norbert Weidner, Rüdiger Rupp

**Affiliations:** Heidelberg University Hospital, Spinal Cord Injury Center, Schlierbacher Landstrasse 200a, 69118 Heidelberg, Germany

## Abstract

**Background:**

For the translation of noninvasive motor imagery (MI)-based brain-computer interfaces (BCIs) from the lab environment to end users at their homes, their handling must be improved. As a key component, the number of electroencephalogram (EEG)-recording electrodes has to be kept at a minimum. However, due to inter-individual anatomical and physiological variations, reducing the number of electrodes bares the risk of electrode misplacement, which will directly translate into a limited BCI performance of end users. The aim of the study is to evaluate the use of focal transcranial magnetic stimulation (TMS) as an easy tool to individually optimize electrode positioning for a MI-based BCI. For this, the area of MI-induced mu-rhythm modulation was compared with the motor hand representation area in respect to their localization and to the control performance of a MI-based BCI.

**Methods:**

Focal TMS was applied to map the motor hand areas and a 48-channel high-resolution EEG was used to localize MI-induced mu-rhythm modulations in 11 able-bodied, right-handed subjects (5 male, age: 23–31). The online BCI performances of the study participants were assessed with a single next-neighbor Laplace channel consecutively placed over the motor hand area and over the area of the strongest mu-modulation.

**Results:**

For most subjects, a consistent deviation between the position of the mu-modulation center and the corresponding motor hand areas well above the localization error could be observed in mediolateral and to a lesser degree in anterior-posterior direction. On an individual level, the MI-induced mu-rhythm modulation was at average found 1.6 cm (standard deviation (SD) = 1.30 cm) lateral and 0.31 cm anterior (SD = 1.39 cm) to the motor hand area and enabled a significantly better online BCI performance than the motor hand areas.

**Conclusion:**

On an individual level a trend towards a consistent average spatial distance between motor hand area and mu-rhythm modulation center was found indicating that TMS may be used as a simple tool for quick individual optimization of EEG-recording electrode positions of MI-based BCIs. The study results indicate that motor hand areas of the primary motor cortex determined by TMS are not the main generators of the cortical mu-rhythm.

## Background

Brain-Computer interfaces (BCIs) are technical systems that provide a direct connection between the human brain and a computer. They are based on the finding that thought-modulated changes in brain activity can be detected and transformed into control signals. Most of the BCI systems rely on bioelectrical brain signals that are recorded noninvasively by electrodes on the scalp (electroencephalogram, EEG). A BCI system consists of five sequential components: (1) signal acquisition, (2) feature extraction, (3) feature translation,(4) classification output, which interfaces to assistive devices, and generates (5) feedback to the user. These components are controlled by an operating protocol that defines the onset and timing of operation, the details of signal processing, the nature of the device commands, and the oversight of performance [1]. At present, EEG-based BCI systems can function in most environments with relatively inexpensive equipment and therefore offer the possibility of practical BCIs in end users’ home environment.

One type of EEG-based BCI exploits the modulation of sensorimotor rhythms (SMRs) in particular the mu-rhythm. The mu-rhythm is an EEG-detectable brain rhythm in the alpha frequency band (8–13 Hz) that can be recorded over the sensorimotor cortex and is most prominent over the representation sites of the hands [2, 3]. While the mu-rhythm is well detectable during a resting state, it can be blocked by execution, imagination [2] or observation [4] of movements, which has led to the description of the mu-rhythm as an “idling rhythm” [5]. The modulation of the mu-band power in response to a motor task has been referred to as event-related desynchronization (ERD) or event-related synchronization (ERS), depending on whether the mu-band power is reduced or enhanced [2]. During a motor task, the cortical representation area of the motor-task-relevant limb experiences an ERD, while surrounding cortical areas often show an ERS. This phenomenon has been referred to as focal ERD/surround ERS [6]. Consequently, motor imagery (MI) tasks of the hand and feet can produce a strong modulation of the mu-band power over the sensorimotor representation area of the hand and have become the custom control tasks for many non invasive SMR-based BCIs [7–9].

One of the main limitations of SMR-BCIs is the problem that in some users the degree of mu-band power modulations does not allow for a sufficient performance of BCI control. In fact, a relevant proportion of users are not able to control an SMR-based BCI with satisfactory accuracy [10]. This inability for controlling a BCI is often called “BCI-illiteracy” [11].

When advancing BCIs towards everyday use, researchers have to restrict the hardware requirements of their systems in order to increase the user-friendliness and applicability [12]. As a key component, the number of EEG-recording electrodes needs to be minimized. However, if only a few electrodes are used to record mu-rhythm modulations, their optimal localization crucially determines the performance of the BCI. Consequently, a BCI system that utilizes a minimal number of EEG electrodes requires an individual optimization of the electrode positions in order to allow a BCI user to exploit her or his full BCI potential.

Only a handful of studies have investigated correlates of inter- and intra-individual variability in BCI performance. Function-based predictors of BCI performance include SMR amplitude at rest [11] and concentration level [13]. Using functional magnetic resonance imaging (fMRI), BCI performance has also been found to correlate with activity in supplementary motor and prefrontal areas during a motor imagery task [14]. Also individual variations in brain or head anatomy might alter the exact localization where the mu-modulation can be optimally recorded [15, 16]. If not compensated for, these inter-individual variations may lead to slight misplacement of the EEG-recording electrodes, which will prevent some BCI users from achieving their top performance and may add to the problem of “BCI-illiteracy”.

High-resolution EEG-measurement in combination with spatial filter algorithms are a valid method for intra-individual determination of optimal electrode positions [17]. However, in a practical setting involving end users with motor impairments the correct placement of up to 64 electrodes is a time-demanding and challenging task. Therefore, an alternative, fast, and easy-to-use method to individually determine the optimal position for the EEG-recording electrodes would be desirable. It has been reported that the cortical areas activated during motor imagery or motor execution tasks show a substantial overlap and involve regions of the sensorimotor cortex [18, 19]. In this respect, the primary motor cortex represents a cortical region that plays a major role in motor execution and can be easily mapped using transcranial magnetic stimulation (TMS). We consequently hypothesized that the hand representation area within the primary motor cortex should be related to the position of the highest mu-rhythm modulation during motor imagery.

Focal TMS is a noninvasive, easy-to-apply method that utilizes a wired coil to produce a powerful and rapidly changing magnetic field, which passes the bony structures of the skull and produces small electrical currents in the region of the brain just under the coil via electromagnetic induction. These currents in turn elicit action potentials in neurons of the targeted area. TMS has become a widely used neurophysiological tool both in clinics as well as in research [20]. Its main clinical applications include the diagnosis and treatment of sensory and motor deficits that arise from neural dysfunction, while in research TMS is mainly used to evaluate corticospinal and intracortical connectivity and plasticity. A clinically relevant measure for corticospinal functionality is the motor evoked potential (MEP). For this, the cortical representation area of a peripheral muscle is stimulated and the corresponding muscular response is measured via electromyography (EMG). The amplitude and latency of the recorded MEP contain information about the functional state of the corticospinal neurons and axons that transmit the signal. Using the amplitude of the MEP as a readout TMS can also be used to map the precise position of the cortical representation area of the recorded muscle with a localization error typically well under 1 cm [21].

In the present study we hypothesized that focal TMS as a clinically established method to map cortical motor representation areas of upper or lower extremity muscles may be used to effectively and quickly determine the optimal electrode position for a noninvasive mu-rhythm-based BCI. Therefore, we compared the positions of the motor hand representation areas of eleven able-bodied subjects with the position of the MI-induced mu-modulation as derived from 48-channel high-resolution EEG recordings during MI tasks of the hands and feet. This design additionally addresses the fundamental question if the cortical primary motor hand area is the main source of mu-band power modulation.

## Methods

The study protocol was approved by the Ethics Committee of Heidelberg University Hospital (approval no. S-626/2013).

### Subjects

Eleven able-bodied, right-handed subjects (5 male, 6 female) with a mean age of 26.6 years (ranging from 23 to 31 years) participated in this study. Handedness of the subjects was determined on the basis of the Hand-Dominanz-Test (H-D-T) [22]. Subjects were excluded in case of active and passive implants and a known history of epilepsy. All gave their written informed consent to study participation. Subjects were paid 10 € per hour. Nine subjects had previous experience (seven had taken part in one 20 min online BCI experiment, one subject had a moderate experience of < 20 online sessions and one subject had high experience > 20 online sessions) with the BCI system while two subjects were BCI-naïve.

### Mapping of the motor hand areas using TMS

**Fig. 1:**
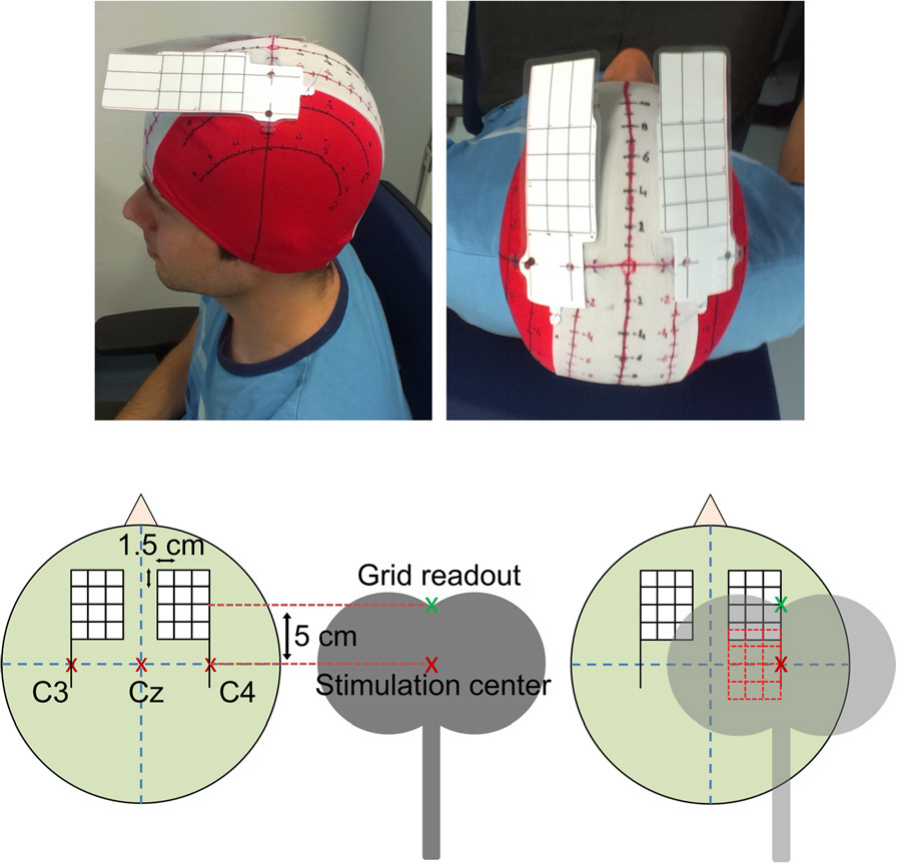
Location of the stimulation sites for the mapping of the motor representation areas of the hand. The stimulation points were marked on a semiflexible grid that was attached to an adherent, elastic cap in such a way that a 6 cm × 4.5 cm area medial to C3 and C4 could be stimulated while the stimulation position could be read out at the upper end of the coil

All subjects sat in a comfortable chair with arm rests. An adherent elastic cap that featured two semi-flexible grids each marking a 6 cm × 4.5 cm stimulation area with 4 × 5 stimulation points at a distance of 1.5 cm [23, 24] in both coordinate directions was placed on their head (Fig. 1).

**Fig. 2:**
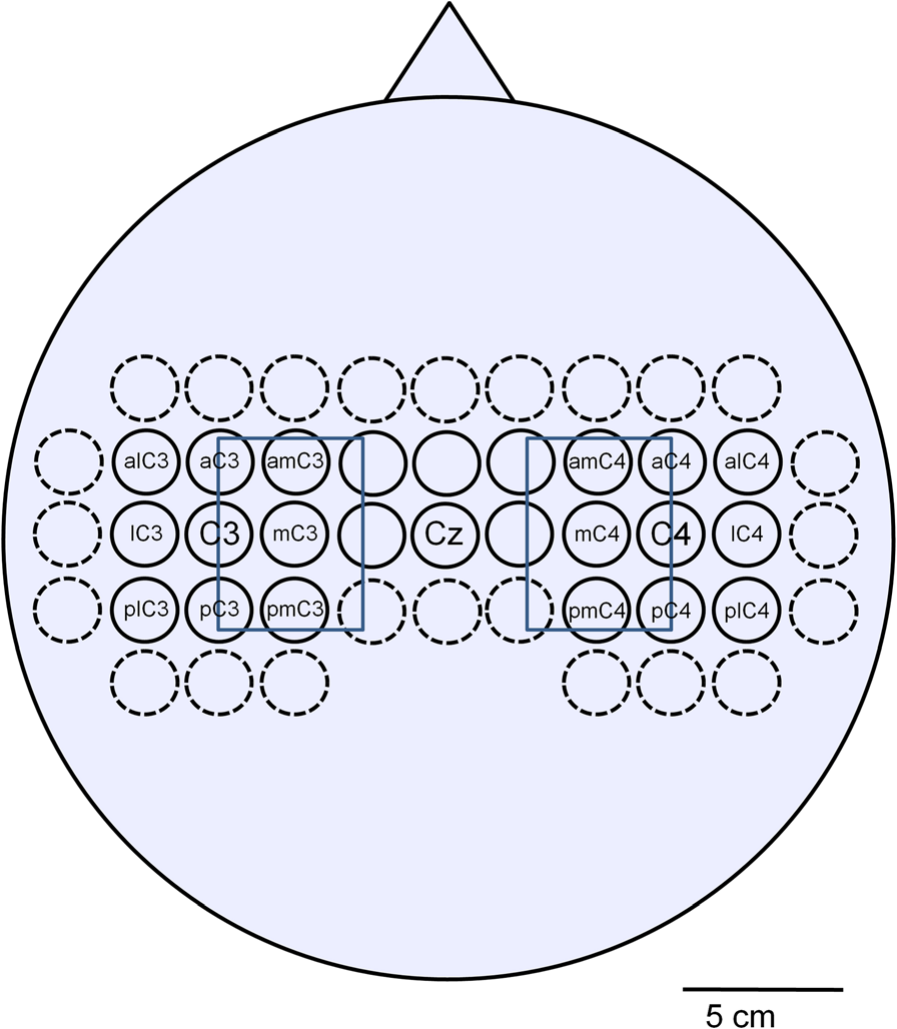
Electrode positions of the 48-channel high resolution EEG recordings. 48 electrodes were placed over the sensorimotor cortex according to a 10–6.7 system with inter-electrode distances of 23 mm in mediolateral and anterior-posterior direction. Electrode positions C3, Cz and C4 are consistent with the international 10–20 system. The central 24 Laplace channels are outlined by solid circles. Electrode positions adjacent to C3 and C4 were annotated according to their use in this paper. In addition, the area of the TMS stimulation grid is overlaid as a blue rectangle

This allowed for the stimulation of a 6 cm × 4.5 cm area medial to C3 (and C4 respectively) that was centered at the C3-C4 axis (Fig. 2). Biphasic stimulation pulses were applied according to the safety guidelines for TMS [25] using a MagPro R100 magnetic stimulator (MagVenture GmbH, Hückelhoven, Germany) and a MC-B70 butterfly-shaped coil (MagVenture GmbH, Hückelhoven, Germany). Both hemispheres were stimulated independently. The amplitudes of the MEPs were recorded from the abductor digiti minimi (ADM) muscle of the contralateral hand using Ambu Neuroline 700 self-adhesive Ag/AgCl electrodes (Ambu A/S, Ballerup, Denmark). The reference electrode was placed on the proximal interphalangeal joint of the little finger. Subjects were advised to relax the recorded muscle during stimulation. In order to find the appropriate stimulation intensity, the stimulator output was stepwise increased starting from 40 % of the maximal stimulator output until reproducible MEP amplitudes greater than 0.5 mV could be elicited at the presumed low-threshold site. The maximum intensity was limited to 70 % to avoid unpleasant sensations. For both hemispheres, each of the 20 stimulations points was stimulated four times in a random order. The butterfly-shaped coil was held parallel to the nasion-inion axis and tangential to the scalp surface with the coil handle pointing posterior. For each hemisphere, the MEP amplitudes of each stimulation point were averaged and expressed as value relative to the highest average amplitude observed. Based on the resulting 4 × 5 MEP amplitude matrix the two-dimensional center of gravity (CoG) was calculated using the equation

$$ {X}_{CoG}=\frac{{\displaystyle {\sum}_{i=1\;}^n\;{a}_i{X}_i}}{{\displaystyle {\sum}_{i=1}^n\;{a}_i}};\;{Y}_{CoG}=\frac{{\displaystyle {\sum}_{i=1}^m\;{a}_i{Y}_i}}{{\displaystyle {\sum}_{i=1}^m\;{a}_i}} $$

Where *a*_*i*_ is the mean MEP amplitude at the site with x-coordinate *X*_*i*_ and y-coordinate *Y*_*i*_ of an n x m MEP amplitude matrix [23, 26]. For each hemisphere, all coordinates including the center of gravity were expressed in terms of their distance to Cz. In order to use the ADM CoG as central location of a nearest-neighbor Laplacian channel during the subsequent online BCI experiments, the ADM CoG was assigned to the electrode position of a 10–6.7 electrode system (EasyCap, Herrsching, Germany) with the shortest Euclidean distance. This electrode position will be referred to as the ADM CoG-nearest electrode. In order to approximate the localization error, the mapping experiment was repeated five times with subject 4 and subject 8. The mean localization error was then derived as the average standard deviation (SD) of the ADM CoG localization of both hemispheres and both subjects. All five mapping experiments were performed within two weeks, but not more than one mapping experiment per day was performed.

### Mapping of the MI-induced mu-modulation with a 48-channel high-resolution EEG

Subjects sat in a comfortable chair with a 15’ screen in front of them. 48 Ag/AgCl sintered ring electrodes (EasyCap, Herrsching, Germany) were placed over the sensorimotor cortex of each subject according to the 10-6.7 system (Fig. 2). In this system, electrode distances were 23 mm in mediolateral and anterior-posterior direction at average head size, thereby representing the electrode grid with the smallest distance available for the EEG system used. In agreement with the international 10–20 system, the lateral distance between Cz and C3 or C4 was 7 cm.

All electrodes were recorded against a reference electrode on the left mastoid and a ground electrode on the right mastoid. At the positions of the electrodes, the scalp was abraded using AbralytHiCl High-chloride abrasive electrolyte-gel (Easycap GmbH, Herrsching, Germany) until all impedances were below 10 kΩ. Electrode signals were recorded with three synchronized g.USBamp USB biosignal amplifiers (G.tec medical engineering GmbH, Schiedlberg, Austria). In order to validate the synchronization between the three amplifiers, a common external clock signal was fed in each of the amplifiers. Before and after each experiment, the recorded clock signal was used to check for sample shifts. Signals were band-pass filtered from 0.5 to 100 Hz and digitized at a sample rate of 512 Hz. The experimental paradigm comprised five motor imagery runs based on the Graz-BCI training paradigm [27]. Each of the motor imagery runs contained 30 trials. Every trial was composed of a two second baseline interval and a five second motor imagery task. The class of motor imagery that the subjects had to perform was indicated by an arrow at the center of the screen that pointed either to the left (demanding a MI of splaying fingers of the left hand), to the right (demanding a MI of splaying the fingers of the right hand) or to the bottom (demanding a feet MI of simultaneously flexing the toes of both feet). Between every two trials a pause interval between 2.5 and 3.5 s was introduced to allow for blinking and swallowing. One run comprised 10 trials of each motor imagery class at a random order, adding up to 30 trials and a duration of 5 min. The EEG data was visually inspected for blink and muscle artifacts. Contaminated trials were excluded.

For the inner 24 electrode channels (Fig. 2, solid circles) the 4 nearest-neighbor Laplacian channel was calculated with equal weights (0.25). For each of the 24 Laplace channels, all trials were grouped according to their class of MI (right hand, left hand or feet). The mu-frequency band (8–13 Hz) was subdivided into four overlapping 2 Hz frequency sub-bands (8–10 Hz, 9–11 Hz, 10–12 Hz and 11–13 Hz) and for every MI class, the mean band power of each sub-band during MI was calculated [28]. Between every two MI classes, the band power difference was calculated and tested for significance using a bootstrap test with 1,000 resamples and a significance level of *p* = 0.05. For those Laplace channels that exhibited significant band power differences, the relative band power modulation between every two classes was calculated as the quotient of the mean band powers of the two MI classes. Channels with nonsignificant band power differences were set to one. Within the 3 × 3 Laplace channel neighborhood around C3 and C4, a mu-modulation center was defined as the electrode position that recorded the largest relative band power modulation between the feet MI and the MI class that involved the represented, contralateral hand (which is the right hand for the C3 neighborhood and the left hand for the C4 neighborhood). If no significant band power differences could be found on the contralateral hemisphere, the most reactive electrode position on the ipsilateral hemisphere was defined to be the mu-modulation center. If none of the hemispheres exhibited a significant mu-modulation, no mu-modulation center was defined. For all subjects the individual distance in mediolateral and anterior-posterior direction between the mu-rhythm modulation center and the ADM CoG was calculated for both hemispheres.

### Control of an online feedback BCI system

The two motor imagery classes that produced the largest relative band power modulation in any of the four mu-frequency sub-bands during the 48-channel EEG measurements were chosen as control tasks for an online 2-class BCI system with continuous visual feedback. For the online BCI trials, the EEG setup was reduced to a single next-neighbor Laplace channel (five electrodes) that was centered either over the control task-specific mu-modulation center or over the ADM CoG-nearest electrode of the same hemisphere. The five electrodes were recorded against a reference electrode on the left mastoid. Again, AbralytHiCl High-chloride abrasive electrolyte-gel (Easycap GmbH, Herrsching, Germany) was used to reduce all impedances below 10 kΩ. Electrode signals were recorded with one g.USBamp USB biosignal amplifier (G.tec medical engineering GmbH, Schiedlberg, Austria) at a sample rate of 512 Hz and using a 0.5–100 Hz band-pass filter. For both electrode arrangements, a linear classifier based on the 48-channel EEG data was calculated as follows: The adjacent frequency sub-bands that showed significant and large relative band power modulations between the control MI classes were combined to a larger, subject-specific frequency band. Based on the mean band power of the combined frequency band of each of the two MI classes a 10 × 10 cross validation was performed to train a Fisher’s linear discriminant with decision boundary 0. For those subjects, for whom the ADM CoG-nearest electrode and the mu-modulation center were identical, only one classifier was calculated. The software to train the classifier was part of the open source BioSig-Toolbox, which is freely downloadable from http://biosig.sf.net/ [29].

**Fig. 3:**
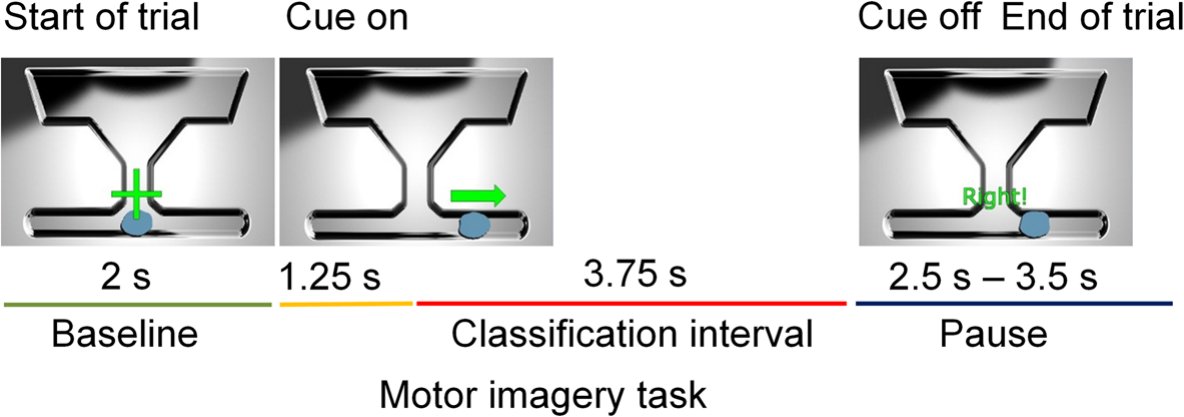
Design of an online BCI trial. One online trial consisted of a two second baseline interval, a five second motor imagery task and a 2.5–3.5 s pause interval. A green arrow, which was continuously shown during the 5 s of the motor imagery interval, indicated the motor imagery class that the subject had to perform. The MI task classification was obtained during the last 3.75 s of the motor imagery task

The online BCI experiment was divided into two blocks that represented the two different electrode arrangements. The sequence of the two blocks was determined randomly for every subject. In each block, the subjects had to perform five online BCI runs that lasted 5 min and comprised 30 trials each. Each trial was composed of a two second baseline interval and a five second MI task (Fig. 3). A green arrow at the start of the MI interval indicated the class of MI that the subject had to perform. This arrow was shown for five seconds throughout the whole MI interval. The classifier output was continuously visualized by the horizontal movement of an animated bubble called liquid cursor display.

The full range of the horizontal bubble movement represented a classifier output range from −1 (most left, representing left hand MI in a left hand vs. feet control task or feet MI in a right hand vs. feet control task) to 1 (most right, representing right hand MI in a right hand vs. feet control task or feet MI in a left hand vs. feet control task). Due to artifacts including eye-movement and visual evoked potentials, the first 1.25 s of the MI interval were not analyzed and only the subsequent 3.75 s interval was used to classify the MI task by integrating the continuous classifier output. Prior to the five online runs, one test run was performed during which the constant bias of the linear discriminant function was manually adjusted if necessary.

The online BCI performance was defined as the percentage of trials where the classification results confirmed the MI class that was indicated at the start of the MI interval i.e., where the subject was able to control the BCI in the appropriate direction. In other words, a trial with a left hand MI was regarded as successful, if the classifier output integrated over the last 3.75 s of the MI interval was negative.

Differences in the online performance between the two electrode arrangements were tested for significance using a two-tailed Fisher’s exact test. For both electrode arrangements the mean online performance of all participating subjects was calculated and differences between both means were analyzed for significance using a paired *t*-test. For those subjects, for whom the ADM CoG-nearest electrode was identical with the mu-modulation center, the online BCI performance based on this common electrode arrangement was assessed and included in both group means.

## Results

On average, subjects completed the mu-modulation center mapping experiment and the online BCI experiment within 5 weeks and never on the same day. The whole set of experiments (transcranial magnetic stimulation, 48-channel EEG measurements and 5-EEG channel online BCI) was on average completed within 125 days ranging from 69 days to 156 days.

### Spatial comparison between the ADM CoG and the mu-modulation center

**Fig. 4:**
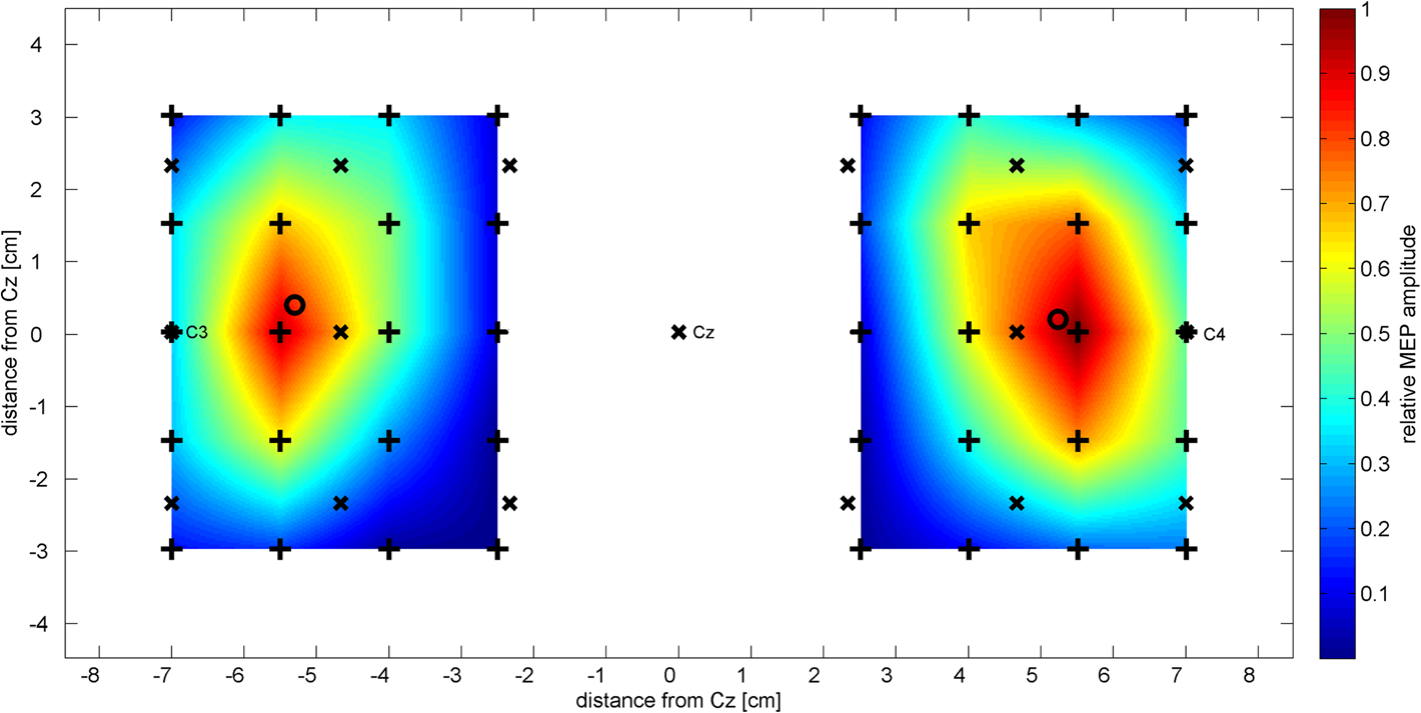
Group average amplitude of motor evoked potentials. The relative MEP amplitudes averaged over all participants are shown for both hemispheres. Stimulation locations are indicated with black crosses, electrode positions of the 10–6.7 system are indicated with a black “x” and the center of gravity for each hemisphere is represented as a black circle. Values between stimulation points are interpolated for better visualization

**Fig. 5:**
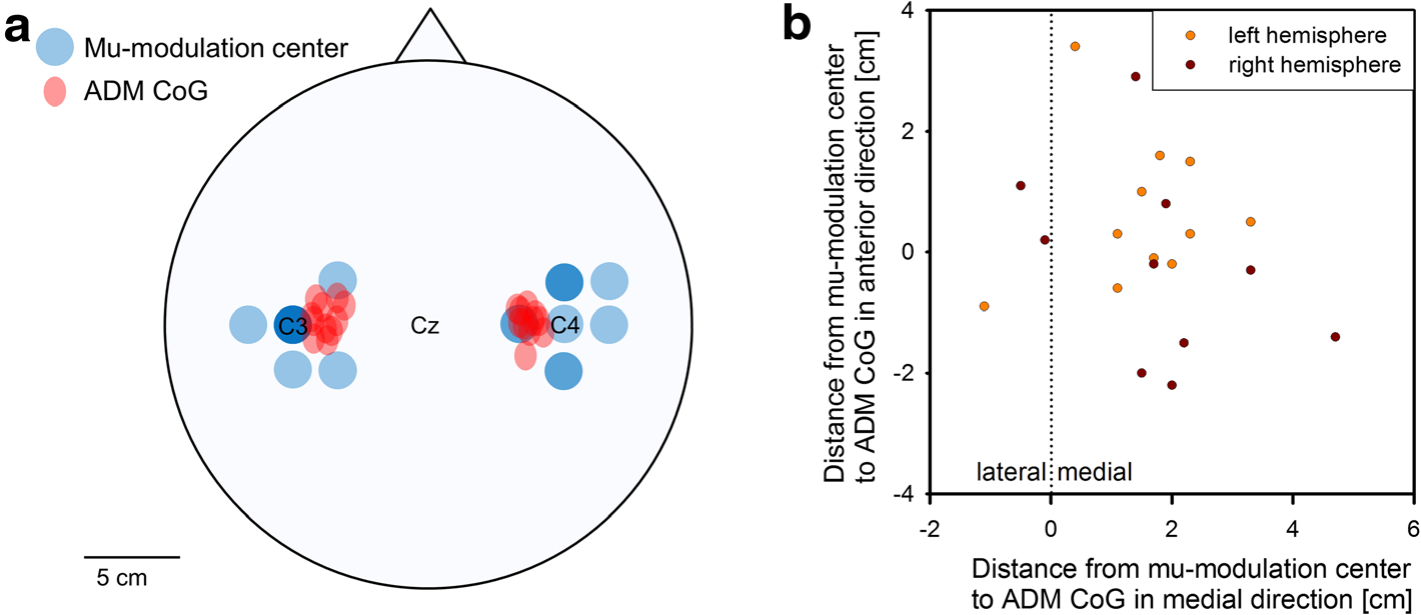
Spatial comparison between ADM CoGs and mu-modulation centers for all subjects and both hemispheres. **a** While the mu-modulation centers (blue areas) are distributed around C3 and C4, the ADM CoGs (red areas) are located more medial. The intensity of the color relates to the number of ADM CoG or mu-modulation centers that overlap at a given position. The size of the red ellipses represents the localization error of the motor mapping in mediolateral and anterior-posterior direction. **b** Individual distances from the mu-modulation center to the ADM CoG in mediolateral and anterior-posterior direction for both hemispheres and all subjects. On average, the ADM CoG was located 1.64 cm medial (SD = 1.30 cm) and 0.31 cm anterior (SD = 1.39 cm) of the mu-modulation center on an individual level

Using focal TMS to map the cortical motor representation area of the ADM muscle, the mean position of the ADM CoG was found to be 5.30 cm (standard deviation (SD) = 0.58 cm) lateral and 0.41 cm (SD = 0.77 cm) anterior to Cz on the left hemisphere and 5.25 cm (SD = 0.44 cm) lateral and 0.21 cm (SD = 0.72 cm) anterior to Cz on the right hemisphere (Fig. 4 and Fig. 5a, red areas).

The mean position of all ADM CoGs (including both hemispheres) was found at a distance of 0.03 cm (SD = 5.42 cm) in mediolateral direction from the inter-hemisphere line. Based on the assumption that the brain is on a group level approximately symmetric to the inter-hemisphere line, this shows that our mapping method was bias-free in mediolateral direction. A localization error of 0.30 cm in mediolateral direction and 0.39 cm in anterior-posterior direction was derived from the repeated mapping of the ADM CoGs of subject 4 and subject 8. This corresponds to 20 % and 26 % of the distance between two stimulation positions in mediolateral and anterior-posterior direction, respectively. Compared to the inter-electrode distance of 23 mm the localization error corresponds to 13 % in mediolateral and 17 % in anterior-posterior direction.

The mapping of the MI-induced mu-modulation using a 48-channel high resolution EEG recording revealed that the mu-modulation centers were distributed around electrode position C3 on the left hemisphere and around electrode position C4 on the right hemisphere (Fig. 5a, blue areas). The electrode position C3 exhibited the strongest relative mu-modulation on the left hemisphere for seven subjects. On the right hemisphere, which represents the left, non-dominant hand, the mu-modulation center was less focused. Here, the strongest mu-modulation could be recorded at electrode positions adjacent to C4 for ten subjects while only one subject had its right hemisphere mu-modulation center at electrode position C4.

For most subjects, a consistent deviation between the position of the mu-modulation center and the corresponding ADM CoG well above the localization error could be observed in mediolateral and to a lesser degree in anterior-posterior direction (Fig. 5b). On average, the ADM CoG was located 1.64 cm medial (SD = 1.30 cm) and 0.31 cm anterior (SD = 1.39 cm) to the mu-modulation center of the same hemisphere on an individual level. Since for subject 9 no significant mu-modulation could be detected on the right hemisphere, only 21 individual distances between the mu-modulation center and the ADM CoG could be calculated (11 for the left and 10 for the right hemisphere).

### Online BCI performance of the mu-modulation center vs. the motor hand area

The electrode positions of the 10–6.7 system that represented the ADM CoG (ADM CoG-nearest electrode) and the mu-modulation center as well as the corresponding online performances and the MI tasks that were used to control the online BCI system are summarized for all subjects in Table 1.

**Table 1:** Laplace channel position, combination of motor imagery tasks and online BCI performance for every subject

Subject	BCI control tasks	ADM CoG-nearest electrode		Mu-modulation center		Significance
		Laplace position	Online performance	Laplace position	Online performance	
5	Right hand vs. feet	mC3	59 %	C3	96 %	***
8	Right hand vs. feet	mC3	87 %	C3	95 %	*
11	Right hand vs. feet	mC3	91 %	C3	92 %	n.s.
2	Right hand vs. feet	mC4	48 %	C4	92 %	***
4	Right hand vs. feet	mC3	65 %	C3	90 %	***
1	Right hand vs. feet	mC3	47 %	pC3	73 %	***
7	Right hand vs. feet	C3	37 %	lC3	72 %	***
6	Left hand vs. feet	mC4	57 %	pC4	64 %	n.s.
3	Right hand vs. feet	amC3	47 %	C3	56 %	n.s.
9	Right hand vs. feet	Identical with mu-modulation center		mC4	53 %	-
10	Left hand vs. feet	Identical with mu-modulation center		amC3	45 %	-
Mean of online performance		-	57.8 %	-	75.3 %	**

Subjects are ranked by their online performance using the mu-modulation center. Most subjects showed a significantly better online BCI performance, when the mu-modulation center was used as the center of a next-neighbor Laplacian channel compared to the motor hand area. Prefixes of Laplace electrode positions: m = 23 mm medial to [electrode position] (Example: mC3 = 23 mm medial to C3), l = 23 mm lateral to [electrode position], a = 23 mm anterior to [electrode position], p = 23 mm posterior to [electrode position] (all electrode positions are also visualized in Fig. 2). Significance levels were *p < 0.05, **p < 0.01, ***p < 0.001 and n.s. *p* > = 0.05

As a consequence of the mediolateral deviation between the mu-modulation center and the ADM CoG, the electrode positions that represented each of the two areas during the online BCI tasks differed by at least one position of the 10–6.7 system for nine subjects. For all of these subjects, the mu-modulation center allowed by trend for a better online BCI performance. For six subjects as well as for the group mean this difference was significant. For subjects 9 and 10 the ADM CoG-nearest electrode and the mu-modulation center were identical, therefore only one common online BCI performance could be assessed. Those subjects with an online BCI performance above 90 % had their mu-modulation center either at C3 or C4, depending on whether the right or the left hand was used in the control task. For two moderately-performing subjects however, the mu-modulation centers that were used to control the online BCI were located at adjacent positions to C3. Most noticeably, for subject 7 the online BCI performance of electrode position C3 was significantly worse than of the electrode position lateral to C3. While the mu-modulation centers of this subject were found particularly lateral (23 mm lateral to C3 for the left and 23 mm anterior and lateral to C4 for the right hemisphere, respectively), the positions of the ADM CoGs were comparable with the group mean. Hence, subject 7 showed the largest mean mediolateral shift of 4 cm over both hemispheres between the ADM CoGs and the mu-modulation centers.

## Discussion

The results from the online experiments confirm well-known knowledge that the major prerequisite for the control of a noninvasive motor imagery-based brain-computer interface is a sufficiently strong modulation of the cortical mu-rhythm band power between two MI tasks. In the high-resolution offline-EEG analysis we found noticeable inter-individual spatial variations of the MI-induced mu-rhythm modulation, which underlines the need for a fast and simple method to individually optimize the positions of the EEG recording electrodes. Based on the individual TMS mapping of the motor hand areas and the locations of the strongest MI-induced mu-modulation, we revealed a clear spatial deviation between the two areas on both hemispheres. This discourages the direct use of the hand motor areas determined by TMS as the optimal EEG-recording electrode positions for a noninvasive SMR-based BCI. However, on an individual level a trend towards a consistent distance between hand motor area and mu-rhythm modulation center was found indicating that TMS may be used to derive an individual offset for optimization of EEG-recording electrode positions in noninvasive mu-rhythm-based brain-computer interfaces. This spatial discrepancy was more consistent in mediolateral direction than in anterior-posterior direction and may need further characterization in order to prove its reliability. In general, all study participants showed a medial shift of the mu-rhythm modulation center in relation to the hand motor area. The largest medial shift was found for subject 7, where the mu-rhythm modulation centers on both hemispheres were located markedly lateral. This may classify subject 7 as a potential outlier and exclusion of data obtained in subject 7 from the overall analysis leads to a less variable mediolateral distance between hand motor area and mu-rhythm modulation center of 1.39 cm (SD = 1.06 cm). This represents a reduction of 26 % of the standard deviation obtained in all subjects.

The localizations of the motor representation sites of the ADM as well as the localization error that we obtained during this study are well consistent with literature [21, 23, 26]. The fact that the variability of the localization of the ADM CoG in our study was generally larger in anterior-posterior direction than in mediolateral direction is most likely due to the oval stimulation area of the butterfly coil that is prolonged in the direction of the handle axis, which in this study was the anterior-posterior axis. The overall location error of the TMS-based motor mapping of the ADM was small compared to the interelectrode distances of the 10–6.7 electrode system. Therefore, the localization of the ADM CoG and its assignment to distinct electrode positions for the online BCI trials can be assumed to be reproducible. In order to further improve the localization accuracy of the TMS-derived motor map, an MRI navigated TMS system may be used [19]. While these systems take into account the individual brain anatomy and physiology and allow for the precise assignment of EEG electrodes to motor map positions, they are usually not available in clinical settings. In the scope of finding a widely-available and easy-to-use tool to position the EEG-recording electrodes of a noninvasive BCI in a patient-orientated setting, nonnavigated TMS appeared to be the more adequate choice.

The electrode positions on the scalp that exhibited the strongest band power modulation within the mu-frequency band were distributed around C3 and C4. Those positions are generally accepted to overly the sensorimotor hand area and to show the strongest event-related desynchronization and synchronization of the mu- and beta-frequency band during motor imagery tasks of the feet and hands [30]. For the control of a MI-based online BCI, a ‘dominant hand’ vs. ‘feet’ paradigm is usually chosen as control task while modulations of the scalp potentials are generally recorded over C3, Cz and/or C4 [10]. The results of this study generally support this approach. For most of our right-handed subjects, a C3 centered Laplace channel combined with a “right hand vs. feet” MI task was found to be most effective to control an online mu-rhythm-based BCI system. However, the general rule, that C3 or C4 provide the best online BCI performance did not apply to two subjects with a moderate BCI performance. Most noticeably, subject 7 showed a significantly better online BCI performance, when the Laplacian channel was centered 23 mm lateral of C3 compared to C3 itself. This indicates that, although the BCI standard positions C3 and C4 provide the best online BCI performance for most subjects, inter-individual variation of the mu-modulation localization exist that justify an individual optimization of the electrode positions in particular at the beginning of a MI-BCI training with initially low to moderate performance.

Since our BCI system operates based on modulations of the mu-rhythm band-power, it was not surprising that the best online BCI performance could be achieved when EEG-recording electrodes were places at the positions of the strongest mu-modulation compared to any other position, including the motor hand area. However, since the position of the strongest mu-modulation was derived from solely offline trials, the evaluation of the online BCI performance represents an important validation in order to quantify the impact of choosing the appropriate electrode positions for online BCI systems during which a user experiences the consequence of its motor imagery strategies directly.

The spatial deviation between the motor hand area and the mu-modulation center found in this study suggests that the primary motor cortex has only a minor role in mu-rhythm generation and modulation. In fact, it is a matter of ongoing debate to what extend the primary motor cortex contributes to motor imagery [14, 31–33]. Functional magnetic resonance imaging and positron emission tomography based studies have failed to draw a consistent conclusion on the involvement of the primary motor cortex during motor imagery tasks. While some of these studies support an involvement during motor imagery [18, 34, 35], others report only minor activation of the primary motor cortex during MI [36, 37]. The strongest support for the role of the primary motor cortex in motor imagery comes from EEG and TMS studies that found that motor imagery increases the excitability of the motor cortex [38] and leads to a change of the cortical rhythms over the sensorimotor cortex [39].

When comparing motor imagery with motor execution, it is assumed that motor execution leads to a stronger activation of the primary motor cortex while motor imagery additionally recruits supplementary motor areas [18, 14]. Since TMS activates mostly the upper motor neurons of the primary motor cortex that project to the spinal cord via the corticospinal tract [40], TMS mapping does not include supplementary motor areas. However, if the spatial deviation between the mu-modulation and the motor representation area of the hand derived solely from the additional recruiting of supplementary motor areas during motor imagery, one would expect that the mu-modulation would rather be localized anterior to the motor representation area. Other studies indicate that the mu-rhythm may be generated more likely in the somatosensory cortex than in the primary motor cortex [41, 42]. In this context, the lateral shift of the mu-modulation centers from the TMS-derived motor hand areas on each hemisphere that was found in this study may reflect a general mediolateral discrepancy between the motor and the somatosensory homunculi that was previously found in electric stimulation [43] and functional magnetic resonance imaging studies [44].

Under the assumption that this mediolateral shift is relatively consistent between individual subjects, the position of the strongest mu-modulation could be derived from the position of the strongest TMS response. This may allow for using TMS as a new, easy-to-apply screening method for individual localization of the optimal electrode positions for recording of the mu-modulation. The findings of this study suggest that the spatial shift between the two positions is a general feature. However, to derive a general positioning rule, further characterization especially in anterior-posterior direction is needed. Future studies involving individuals with high spinal cord injuries and the associated loss of motor and sensory function of feet and hands may provide additional insights into the physiological mechanisms of the generation of the mu-rhythm.

The use of nonnavigated transcranial stimulation to map the cortical motor representation area of the hand represents a technical limitation of this study. When compared to navigated MRI-supported TMS, non-navigated TMS has a reduced accuracy and reliability [21, 23, 26]. However, in the light of the main purpose of this study there was no alternative to nonnavigated TMS due to its easiness of use and its wide clinical availability.

## Conclusions

EEG-recordings over the mu-modulation center allowed for a significantly better online-BCI performance than EEG-recordings over the motor hand area determined by TMS. On an individual level a trend towards a consistent spatial distance between motor hand area and mu-rhythm modulation center was found indicating that TMS may be used as a simple tool for quick individual optimization of EEG-recording electrode positions in noninvasive mu-rhythm-based brain-computer interfaces. In our study the spatial relation between motor hand area and mu-rhythm modulation center was less consistent in anterior-posterior direction than in mediolateral direction and should be further characterized. Being spatially distinct from the cortical areas of greatest mu-modulation, the motor hand areas of the primary motor cortex determined by TMS can be assumed to be not the main sources of the mu-rhythm generation.

## Abbreviations

ADM: Abductor digiti minimi muscle
BCI: Brain-computer interface
CoG: Center of gravity
EEG: Electroencephalogram
ERS/ERD: Event related synchronization/event related desynchronization
MEP: Motor evoked potential
MI: Motor imagery
SD: Standard deviation
SMR: Sensorimotor rhythm
TMS: Transcranial magnetic stimulation
